# Complete genome sequence of *Azospira* sp. strain I13 harboring two distinct nitrous oxide reductase (*nosZ*) genes

**DOI:** 10.1128/mra.00925-25

**Published:** 2026-01-20

**Authors:** Younha Ok, Shohei Yasuda, Takahiro Ogawa, Aoi Hosaka, Masafumi Yohda, Akihiko Terada

**Affiliations:** 1 Department of Applied Physics and Chemical Engineering, Tokyo University of Agriculture and Technology 155204 , Koganei, Tokyo, Japan; 2 Civil Engineering, College of Science and Engineering, University of Galway 8799, Galway, Ireland; 3 Department of Biotechnology and Life Science, Tokyo University of Agriculture and Technology 13125, Koganei, Tokyo, Japan; 4 Nihon BioData Corporation, Kawasaki, Kanagawa, Japan; University of Southern California, Los Angeles, California, USA

**Keywords:** denitrifying bacteria, nitrous oxide, *nosZ*

## Abstract

We report on the complete genome sequence of *Azospira* sp. strain I13, a class of *Betaproteobacteria*. We identified two distinct nitrous oxide (N_2_O) reductase (*nosZ*) genes in the retrieved genome. Elucidating the genetic repertoire may clarify the high N_2_O-reduction activity and guide novel mitigation technologies.

## ANNOUNCEMENT

N_2_O reductase (NosZ) reduces N_2_O to N_2_ in the final denitrification step, functioning as a N_2_O sink (1, 2). *Azospira* sp. strain I13 isolated from a bioreactor using N_2_O as the sole electron acceptor (3) is a clade II *nosZ*-type N_2_O reducer with high reduction rate and affinity (4). Although the draft genome offered initial metabolic insights (1), fragmentation may have obscured key features. Hence, this study reports the complete genome of *Azospira* sp. strain I13 for metabolic reconstruction and future comparative genomics.

*Azospira* sp. strain I13 was revived from a glycerol stock and cultured aerobically on JCM Medium No. 12 agar (pH 7.5, 30°C) for 3 days, after which a single colony was subcultured under the same conditions. Genomic DNA for Illumina short-read sequencing and high-fidelity (HiFi) long-reads sequencing was extracted separately using ISOIL for Beads Beating (Nippon Gene, Japan) and NucleoBond HMW DNA kits (Macherey-Nagel, USA), respectively, following the manufacturers' protocols. DNA for Illumina sequencing was purified using RNase A treatment, followed by phenol-chloroform extraction, whereas DNA for HiFi sequencing was purified with RNase A treatment alone. The HiFi DNA samples were used to construct multiplexed amplicon libraries with the SMRTbell Express Template Preparation Kit 2.0 (PacBio, USA), and no size selection was applied prior to long-read sequencing on the Sequel IIe System (PacBio, USA). The Illumina DNA samples were used to construct Illumina libraries with the NEB Next Ultra II DNA PCR-Free Kit (E7410) and sequenced on the Novaseq 6000 platform (Illumina, USA). HiFi sequencing yielded 94,237 reads (0.84 Gbp), and Illumina short-read sequencing generated 7.5 million paired-end reads (1.1 Gbp). Both data sets were used for downstream analyses, and the Illumina and HiFi reads were combined for subsequent processing (Table 1).

**TABLE 1:** The sequence results of *Azospira* sp. strain I13

PacBio HiFi reads							
Number of reads	Total bases (bp)	Coverage	Minimum read length (bp)	Maximum read length (bp)	Mean read length (bp)	N50	Ns(%)
94,237	835,961,117	217´	791	35,037	8870.84	10,626	0
**Illumina Short reads**							
**Number of reads**	**Total bases (bp)**	**Coverage**	**Read length (bp)**			**Q30 bases (%)**	
7,498,282	1,124,742,300	292´	150			94	

Long reads were mapped to the draft genome of *Azospira* sp. strain I13 (NCBI, GCF_003112695.1) using minimap2 v2.24 (5). The filtered reads were assembled with Flye v2.9.2 (6) and circularized using Circlator v1.5.5 (7). Short reads were quality-trimmed with fastp v0.23.2 (8) (Q30 cutoff) and used to polish the assembly with bwa-mem2 v2.2.1 (9) and Pilon v1.23 (10). Assembly completeness was evaluated with BUSCO v5 (11) (bacteria_odb10) via gVolante v2.0.0 (12), resulting in 100% completeness and 0.8% duplication. Circularization was further supported by the absence of terminal overlaps in MUMmer v4.0.1 (13) self-alignment. The polished assembly was visualized using gfastats v1.3.11 (14) and Bandage v0.8.1(15), showing a single-node topology.

The final assembly comprised a single circular chromosome (3,858,033 bp; GC content, 63.8%) with no gaps and an estimated 217× HiFi read coverage. Genome annotation using DFAST v1.2.17 (16) and BlastKOALA v3.1 (17) identified 3,441 CDSs (average length, 335 amino acids; coding ratio, 89.7%), eight rRNAs, and 70 tRNAs. Notably, the complete genome harbors two distinct *nosZ* genes.

## Data Availability

The accession numbers of whole-genome sequence AP044851, BioProject PRJDB35888, BioSample SAMD01608793, Experiment DRX698909-DRX698910, and Run DRR718888–DRR718889 have been deposited in DDBJ/NCBI/EMBL.
